# CO_2_ Mitigation Potential of Plug-in Hybrid Electric Vehicles larger than expected

**DOI:** 10.1038/s41598-017-16684-9

**Published:** 2017-11-28

**Authors:** P. Plötz, S. A. Funke, P. Jochem, M. Wietschel

**Affiliations:** 1 Fraunhofer Institute for Systems and Innovation Research ISI, Breslauer Strasse 48, 76139 Karlsruhe, Germany; 2 Institute for Industrial Production (IIP), Chair of Energy Economics, Karlsruhe Institute of Technology (KIT), Hertzstraße 16, Building 06.33, 76187 Karlsruhe, Germany

## Abstract

The actual contribution of plug-in hybrid and battery electric vehicles (PHEV and BEV) to greenhouse gas mitigation depends on their real-world usage. Often BEV are seen as superior as they drive only electrically and do not have any direct emissions during driving. However, empirical evidence on which vehicle electrifies more mileage with a given battery capacity is lacking. Here, we present the first systematic overview of empirical findings on actual PHEV and BEV usage for the US and Germany. Contrary to common belief, PHEV with about 60 km of real-world range currently electrify as many annual vehicles kilometres as BEV with a much smaller battery. Accordingly, PHEV recharged from renewable electricity can highly contribute to green house gas mitigation in car transport. Including the higher CO_2eq_ emissions during the production phase of BEV compared to PHEV, PHEV show today higher CO_2eq_ savings then BEVs compared to conventional vehicles. However, for significant CO_2eq_ improvements of PHEV and particularly of BEVs the decarbonisation of the electricity system should go on.

## Introduction

The limited range of battery electric vehicles (BEV) is a major factor impeding the mass market diffusion of BEV^1^. Only a decreasing battery price together with an increasing range of vehicles and a reliable fast charging system might overcome this issue. Presently, plug-in hybrid electric vehicles (PHEV) that combine an electric drive train with a conventional one provide already a suitable technology for all mobility patterns. The propulsion of a PHEV can be provided by two modes: In charge depleting mode only the electric engine is operating. In charge sustaining mode (usually applied when the battery has been depleted), the combustion engine is providing the energy for propulsion and keeps the battery state-of-charge within a small window. For some PHEV models, also blended modes exist where both the combustion engine and the electric motor are working in parallel following complex schedules.

Increasingly ambitious limitations on CO_2_ fleet emissions are based on official driving cycles and applied in most regions of the world^2,3^. They have a significant effect on carmakers and vehicle users. The current consideration of PHEV in these driving cycles is somewhat arbitrary and emissions of electricity production are neglected^4^. Many countries give higher purchase subsidies to BEV since they show no direct CO_2eq_ emissions during their use phase^4^. However, to favour BEV over PHEV requires higher contributions to greenhouse gas mitigation in real-world conditions. Consequently, an empirical evaluation of both BEVs’ and PHEVs’ climate contribution is of high value for policy makers, car industry and potential users.

From an analytical point of view, PHEV fuel consumption depends on the PHEV’s all-electric range (AER) and the typical distance driven between recharging. The utility factor (UF) is the share of electrified kilometres of total kilometres driven of a PHEV^5,6^. Assessing empirical fuel consumption of PHEV is challenging since the share of electricity and conventional fuel for propulsion is never measured officially and strongly depends on the individual driving and charging patterns.

## Results

### Real-world electric driving shares

**Figure 1:**
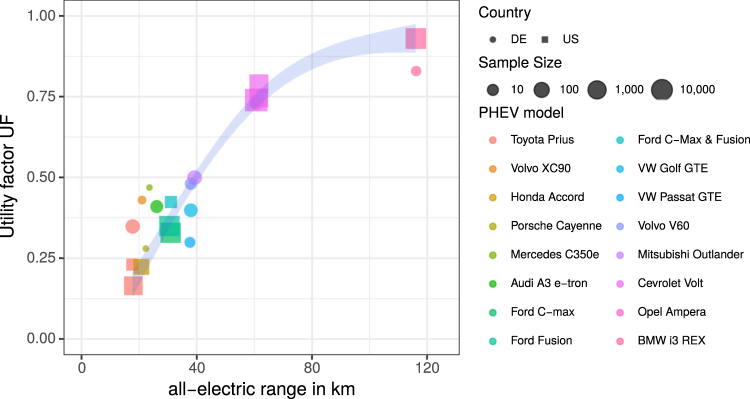
Real-world utility factors of PHEV in the US (squares) and Germany (circles) with different AER. Shown are mean values per PHEV model sorted by increasing AER with the symbol size indicating the size of the sample as well as a sample size weighted local regression (shaded area). We use EPA AER for US models and 75% NEDC AER for German models.

Here, we analyse average UF from 73,000 PHEV covering 16 different PHEV models driven in the US and Germany. Our data has been collected from vehicle usage monitoring websites (voltstats.net and spritmonitor.de) as well as data collections from fleet tests by US research institutes (UC Davies and Idaho National Lab) and car manufacturers^7,8^. Real-world UF depend strongly on the AER and thus differ considerably between PHEV models (cf. Figure 1). The typical AER of current PHEV models amounts to about 50 km in the New European Driving Cycle (NEDC) – currently applied in Europe and China. Note that real-world AER are often shorter than the official NEDC ranges since real-world energy consumption of passenger cars is currently about 30–40% higher than NEDC values^9^. The higher energy consumption in real-world usage also leads to smaller UF than expected from NEDC values (cf. Figure A-2 in the supplementary materials). The NEDC estimate for the UF is based on norm ECE R101^10^ and given by UF_NEDC_ = AER/(AER + 25 km). Several studies measure real-world UF for various PHEV models over the recent years. In the US, AER are obtained from EPA testing procedures and published by the US Department of Energy^11^. EPA testing procedures are usually more stringent than NEDC and lead to about 25% smaller AER (when comparing models that are available in both markets).

Combining the US and German data one obtains distributions of real-world UF for different AER. Figure 1 shows the average UF for different PHEV models in the US and Germany. We use the EPA AER for the US models and 75% of the NEDC AER for German PHEV models.

A local regression shows that about 30% UF can be expected for a 25 km AER that increases to almost 50% for 40 km AER (corresponding to 50 km NEDC AER). Beyond that range, the gain in UF per additional km of AER lessens. Furthermore, the difference between real-world and test-cycle UF decreases with growing AER (cf. Figure A-1 in the supplementary materials).

Despite the AER, several factors influence an individual PHEV’s UF. The re-charging frequency and the distribution of driving distances can significantly affect the individual UF such as recharging at work or during stops of long-distance driving. For example, if company car drivers have to pay for electricity at home but do not for conventional fuel, they show much lower average UF than private car buyers^12^. Furthermore, many long-distance trips without recharging lead to a lower UF and are correlated with high annual-mileage. Thus very high annual vehicle kilometres travelled (VKT) leads to lower average UF^13^.

### Travel Distance Electrification

**Figure 2:**
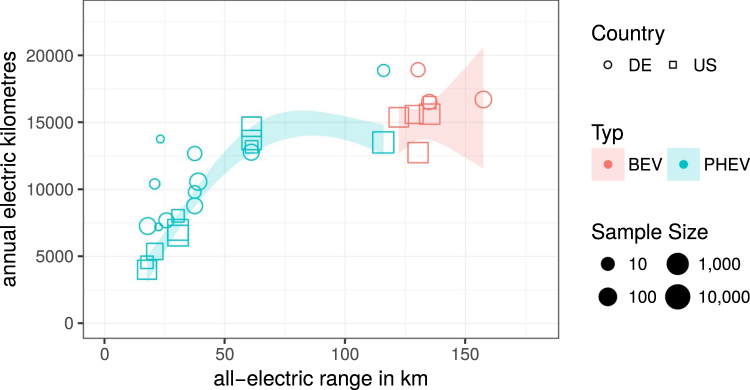
Average electrified annual kilometres for different PHEV (green) and BEV (red) models from the US (squares) and Germany (circles). The shaded areas are sample size weighted local smoothers (95% confidence bands).

Based on the actual UF in different markets we can compare the average annual kilometres electrified by PHEV and BEV in Germany and the US (cf. Figure 2). The sample comprises 73,000 PHEV and 49,000 BEV from the US and Germany. For PHEV the total annual VKT have to be combined with the UF to obtain the electric annual VKT. For BEV, the total annual VKT is identical to the electric annual VKT. All models show high annual driving with an average annual VKT of 21,700 km for the PHEV and 18,400 km for BEV compared to annual averages of 18,600 km for the US^14^ and 14,300 km for Germany^15^. Per PHEV model averages range from 14,500 to 32,700 km and per BEV model averages range from 12,700 to 34,600 km (for Tesla Model S – not shown in Fig. 2).

**Figure 3:**
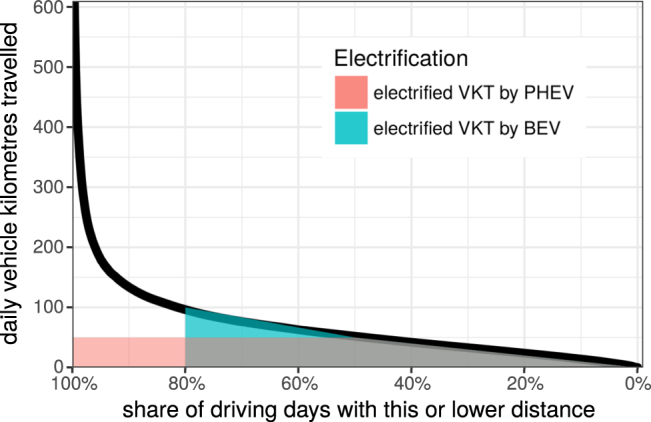
Overall distribution of daily VKT for a large daily driving data set. Also shown are the annual electrified VKT by BEV and PHEV with typical ranges as shaded areas under the curve.

Not surprisingly, the average number of annually electrified kilometres increases with AER. Figure 2 shows that long-ranged PHEV with AER around 60 km achieve about 12–15,000 electrified VKT similar to BEV with about 12–17,000 km. But a 60 km PHEV requires only about half the battery capacity for this electric mileage. Accordingly, battery usage as measured in annual electrified VKT per kilometre of AER is much higher for PHEV. While PHEV achieve an average of 218 electrified VKT per km of AER, BEV yield about 108 km of electrified VKT per km of AER. Thus, for a given battery size PHEV electrify about twice as much of their annual VKT. The basic reason is that PHEV electrify a noteworthy share of every day’s VKT whereas BEV electrify all VKT on some days only (cf. Figure 3).

The high utilisation of PHEV batteries compared to BEV batteries can be illustrated by the distribution of individual daily driving distances^16^. Figure 3 shows the distribution of all daily VKT extracted from the full sample of the voltstats.net database (1,738 PHEV, thick black line). The vehicles are all Chevrolet Volt PHEV from  North America with an average annual VKT similar to the US average. Typically, about 80% of the daily VKT are below 100 km and well within BEV ranges. The electrified VKT by BEV and PHEV are illustrated as shaded polygons in Fig. 3 with assumed BEV range of 100 km and AER of 50 km for a PHEV. Yet, the areas of both polygons are similar: the BEV triangle has an area of ½ · 80% · 100 km · 365 = 14,600 electrified VKT and the PHEV polygon is a small triangle ½ · 50% · 50 km · 365 plus a larger rectangle 50% · 50 km · 365 summing up to 13,700 electrified VKT. Both numbers for total electrified VKT are in line with typical values from Fig. 2.

In the future, when more fast charging stations will be available, BEV are going to increase their electrified VKT significantly as more trips will become feasible with inter-mediate charging. This implies that more long range trips could be electrified by BEV (the green triangle in Fig. 3 grows to the left). In principle, PHEV could also recharge during long-distance trips but the small AER requires many breaks for recharging and users might prefer to refuel their fuel tank instead. Yet this also depends on the dimensioning of battery and tank capacities of PHEV.

### Impact on Greenhouse Gas Emissions

The higher utilisation of the given battery has implications for the life cycle CO_2eq_ emissions of PHEV as compared to BEV. While the current emissions during the vehicle construction are higher for BEV (due to the higher battery capacity), the higher emissions during the vehicle usage phase by the combustion engine is balancing this PHEV advantage.

Today, one kWh of battery capacity causes about 100 kg CO_2eq_ emissions during its construction phase^17^ (see also in the supplementary materials). Consequently, the additional construction phase emissions for the battery are smaller for PHEV (on average 0.6 t of CO_2eq_) than for BEV (on average 2.6 t of CO_2eq_). Yet, PHEV include internal combustion engines and complex gear boxes, which lead to additional emissions of about 0.6 t of CO_2eq_ per vehicle during the construction phase (see supplementary materials).

Thus, currently the overall CO_2eq_ emissions from vehicle construction are about 1.4 t higher for BEV than for PHEV. Learning effects in battery production together with an improved electricity mix might even decrease this disadvantage in the future (cf. Figure A-5 in the supplementary materials).

**Figure 4:**
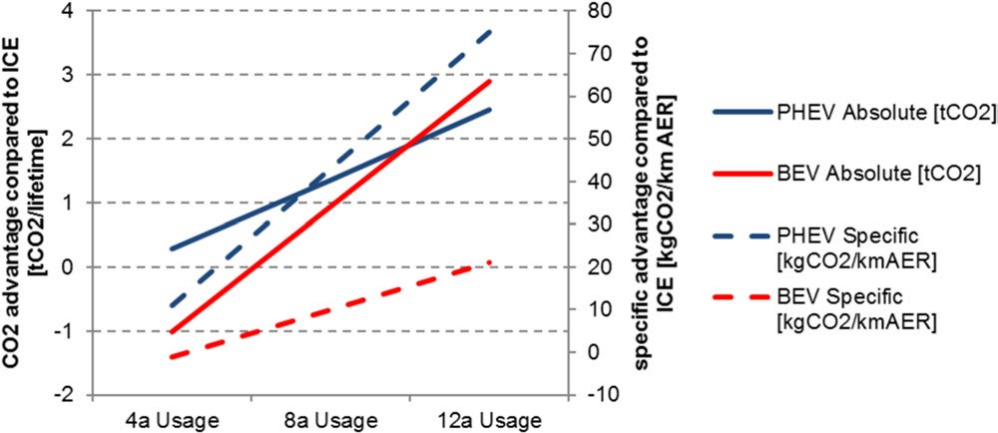
Lifecycle advantages of CO_2eq_ emissions from PHEV and BEV compared to conventional vehicles on an absolute scale and relative to battery capacity.

During the vehicle usage phase BEV might outweigh their disadvantage if their VKT is high and electricity causes few CO_2eq_ emissions. For keeping our results comparable and comprehensible, we take for both countries average emissions values for electricity generation of 500 gCO_2eq_/kWh (c.f. Figure A-4 in the supplementary materials). The gross specific energy consumption for electric driving is assumed to be 0.20 kWh/km (see SM). This leads to an emission factor of 100 gCO_2eq_/km in charge depleting mode, while we assume for the charge sustaining mode a real world emission factor of 120 gCO_2eq_/km (corresponding to 4.6 l/100 km). Over lifetime the overall CO_2eq_ emissions of our PHEV and BEV under investigation show similar values. When assuming short life times, the PHEV is advantageous, while for longer vehicle life time (above 10 years) the BEV becomes more attractive. We assume that the lifetime of the battery equals the lifetime of the vehicle (even though this might be optimistic for our assumed usage phase of 12 years). The overall emission reductions from EV compared to conventional vehicles amount in average to 0.6 t of CO_2eq_ for PHEV and 0.2 t of CO_2eq_ for BEV for eight years of vehicle life times; for life times below 4 years, the BEV advantage to conventional cars becomes even negative (c.f. Figure 4). Per battery capacity, the CO_2eq_ advantage from PHEV at the current electricity mix emissions exceeds those from BEV significantly. For a twelve-year vehicle life time the advantage for our global climate per battery capacity is almost fourfold (i.e. 43 vs 12 kg CO_2eq_ per km AER, see Fig. 4).

The specific emissions from the electricity grid are very decisive. An average emissions value of 470 gCO_2eq_ per kwh (i.e. 6% improvement) outweighs already the advantages for PHEV for the eight-year life time scenario. Hence in all American states with better specific emission levels than Alabama (c.f. Figure A-4 in the supplementary materials), BEV are already favourable in our scenario setting. In the future, when the electricity generation decarbonizes and the emission during battery production decline, the BEV will become more and more advantageous for reducing greenhouse gas emissions. In 2030, where the energy transition reduced specific emissions in Germany down to about 290 gCO_2eq_ per kWh^18^, the advantage of EV will be preeminent: during an eight-year life time the absolute gain in CO_2eq_ emissions compared to improved conventional vehicles with average real-world emissions of 100 gCO_2eq_ per km, amounts to 2.2 t of CO_2eq_ for PHEV and 3.1 t of CO_2eq_ for BEV. This corresponds to specific advantages of 69 kg of CO_2eq_ per km AER for PHEV and 20 for BEV. Hence, while for green electricity the absolute emissions for BEV becomes very favourable, the PHEV can still outperform in terms of specific emissions for per km AER for current driving profiles.

## Summary and Conclusions

PHEV are an alternative to BEV in terms of electrifying mileage in order to mitigate GHG emissions from transport. However, little is known about their average UF and annual electrified mileage in real-world driving. Here, we present the first systematic overview on usage of PHEV in comparison to BEV with a total sample of 73,000 PHEV and 49,000 BEV from the US and Germany. We find the average UF is mainly dependent on the all-electric range (AER) and ranges from 15–35% for about 20 km, about 40–50% for 40 km and about 75% for about 60 km of real-world AER. Today, empirical mobility patterns show that PHEV with about 60 km of AER electrify about the same annual mileage with half the battery capacity compared to BEV. A suitable fast charging system might however reverse this advantage. Currently, EV decrease CO_2eq_ emissions compared to conventional vehicles and for vehicle life times below about 7 years, the effect from PHEV dominates today. This is a fortiori true for the specific emission reductions based on battery capacity. A decarbonisation of electricity generation will improve the CO_2eq_ emissions from BEV considerably.

## Methods

### Electric vehicle data

Voltstats.net is an online database that collects real-world fuel economy performance data of Chevrolet Volt, mainly in the U.S., with more than 1,800 reported Chevrolet Volt driven in the US and Canada (voltstats.net)^8^. It comprises data from registered users with a comprehensive set of user specific performance data (see the supplementary materials for more detailed information). Based on the available data we calculated the following parameters: The average total monthly miles were extrapolated to annual mileage. The individual UF is obtained by dividing all electric miles by total miles driven. The individual total fuel consumption is the product of fuel consumption in charge sustaining mode and the share of conventional driving, i.e. 1-UF.

We based our analysis on German PHEV driving on Spritmonitor.de, a German online web service for car drivers to calculate real-world kilometre cost including all operating cost. This database contains information for different vehicle types, including PHEV. Among other information, registered car drivers report their fuel demand in litres and the corresponding cost as well as the vehicle mileage after each refuelling. The resulting average fuel consumption and cost are calculated automatically. Detailed information on distances travelled and the respective fuel consumption for every registered driver are accessible freely on the website. Mock *et al*.^9^ indicate a good representativeness of spritmonitor.de for the German car fleet.

### Statistical methods

All calculations and the local weighted regression analysis has been performed with the R statistical language^19^. More specifically, the local regression lines are locally weighted scatterplot smoothers (LOESS and GAM).

### GHG emissions impact

For estimating the climate impact from electric vehicles two crucial assumption hast to be made. The first is on the emissions of GHG during the production process of the battery and the second is on the caused, indirect GHG emissions during the vehicle usage phase. The production process of batteries is complex^20^ and run through significant improvements during the last years but is still far from being completely matured. This development is already acknowledged from literature and the average emission values are still between 39–196 kg CO_2eq_/kWh^17^. Correspondingly, we assumed for our calculation an additional emission value of 18 kg of CO_2eq_ per km AER.

However, PHEV still have an internal combustion engine and complex gear box, which is associated with a more complex production process, than an electric motor without a gear box. For current passenger cars the share of emissions of CO_2eq_ during the vehicle production phase for the engine and gear box amount to about 20%^21^. The emissions for the production of an electric motor are still uncertain. We therefore assume the additional emissions for the internal combustion engine of PHEV of about 0.6 t of CO_2eq_ per vehicle. Hence, the CO_2eq_ emissions during the production phase for BEV is reduced by 0.6 t of CO_2eq_ compared to ICEV and PHEV.

### Data Availability

All data generated or analysed during this study are included in this published article (and its Supplementary Information files).

## Electronic supplementary material

Supplementary Material
