# Supplementary material for: CO2 Mitigation Potential of Plug-in Hybrid Electric Vehicles larger than expected
Source: Sci Rep. 2017 Nov 28;7:16493. doi: 10.1038/s41598-017-16684-9 (PMC5705705; doi:10.1038/s41598-017-16684-9)
Supplement: Supplementary file 1 — Supplementary Material [file 41598_2017_16684_MOESM1_ESM.pdf]

CO_2 Mitigation Potential of Plug-in Hybrid Electric Vehicles larger than expected - Supplementary Material to

P. Plötz[1*], S. A. Funke[1], P. Jochem[2], M. Wietschel[1]

[1] Fraunhofer Institute for Systems and Innovation research ISI, Breslauer Strasse 48, 76139 Karlsruhe, Germany

[2] Institute for Industrial Production (IIP), Chair of Energy Economics, Karlsruhe Institute of Technology (KIT), Hertzstraße 16, Building 06.33, 76187 Karlsruhe, Germany

* corresponding author: patrick.ploetz@isi.fraunhofer.de, phone: +49-721-6809289

US PHEV and BEV data

Our analysis on US PHEV driving is based on two large datasets, voltstats.net and CARB (2017)[1]. Both datasets comprise more than 30.000 observations of six different PHEV models.

Voltstats.net is an online database that collects real-world fuel economy performance data of Chevrolet Volt, mainly in the U.S., with 1,738 reported Chevrolet Volt driven in the US and Canada (voltstats.net). It comprises data from registered users with a comprehensive set of user specific performance data (see also Table A-1). The average number of days observed per vehicle is 442 days with a minimum of 17, median of 382 and maximum of 1,327 days. On the basis of the available data we calculated the following parameters: The average total monthly miles were extrapolated to annual mileage. The individual UF is obtained by dividing all electric miles by total miles driven. The individual total fuel consumption c_{tot} is the product of fuel consumption in charge sustaining mode c_{cs} and the share of conventional driving, i.e. $1 - \text{UF}$.

Table A-1: Description of the PHEV database voltstats.net.

	**voltstats.net**
Available Data	Total miles, electric miles, different fuel economy values, residence
Derivable data	Annual mileage, utility factor
PHEV Model	Chevrolet Volt ($N = 1{,}738$)
Data collection	Collected via interface to OnStar (telematic system)
Data availability	2012-2014
Fleet structure	Mainly private cars

An overview of several empirical PEV studies has been compiled in CARB (2017)[1]. It summarises average PEV annual VKT and UF from fleet tests by car manufacturers and US research institutes (UC Davis (UCD) and Idaho National Lab (INL)). A summary of the different PHEV models is given in Table A-2 and all summary statistics in Table A-3.

Table A-2: Overview of the North American PHEV available in CARB (2017).

**PHEV Model**	**All-electric range* [km]**	**Test-cycle UF***
Toyota Prius PHEV	18	29%
Honda Accord	21	33%
Ford C-Max Energi	31	45%
Ford Fusion Energi	31	45%
Chevrolet Volt	61	68%
BMW i3 REX	116	83%

*US-EPA testing, c.f. https://www.fueleconomy.gov/feg/fe_test_schedules.shtml

Table A-3: Summary statistics of North American PEV model data as collected by the authors and reported in CARB (2017) [1].

Model	PEV type	Source	Sample size	All-electric range* [km]	Empirical UF	Electric VKT in km	Electric VKT per km AER
Prius PHEV	PHEV	INL	1,523	18	16.4%	3,998	414
	PHEV	UCD	18	18	23.1%	4,553	472
Ford C-max	PHEV	INL	5,368	31	32.8%	6,548	214
Ford Fusion	PHEV	INL	5,803	31	35%	6,980	228
Ford C-Max & Fusion	PHEV	UCD	18	31	42.3%	8,018	262
Chevrolet Volt	PHEV	INL	1,867	61	74.5%	14,664	240
	PHEV	UCD	18	61	73.6%	13,174	215
	PHEV	Plötz et al. (2017)[13]	1,738	61	79%	13,676	224
	PHEV	GM	48,000		74%	NA	NA
Honda Accord	PHEV	INL	189	21	22.3%	5,369	257
BMW i3 REX	PHEV	CARB	8,309	116	93%	13,498	116
PHEV TOTAL			72,944				
Nissan Leaf	BEV	INL	4,038	135	100%	15,606	115
	BEV	UCD	18	135	100%	16,464	122
Ford Focus electric	BEV	INL	2,196	122	100%	15,366	126
Honda Fit	BEV	INL	645	129	100%	15,578	121
Tesla Model S	BEV	CARB	37,635	335	100%	21,716	65
BMW i3	BEV	CARB	4,193	130	100%	12,740	98
BEV TOTAL			48,725				

*US-EPA testing, c.f. https://www.fueleconomy.gov/feg/fe_test_schedules.shtml

The following Figure A-1 summarises the results of the UF of all US PHEV as function of all electric range.

[1] Mainly Table 9 in CARB (2017) as well as tables 11 and 20 in Appendix G of CARB (2017).

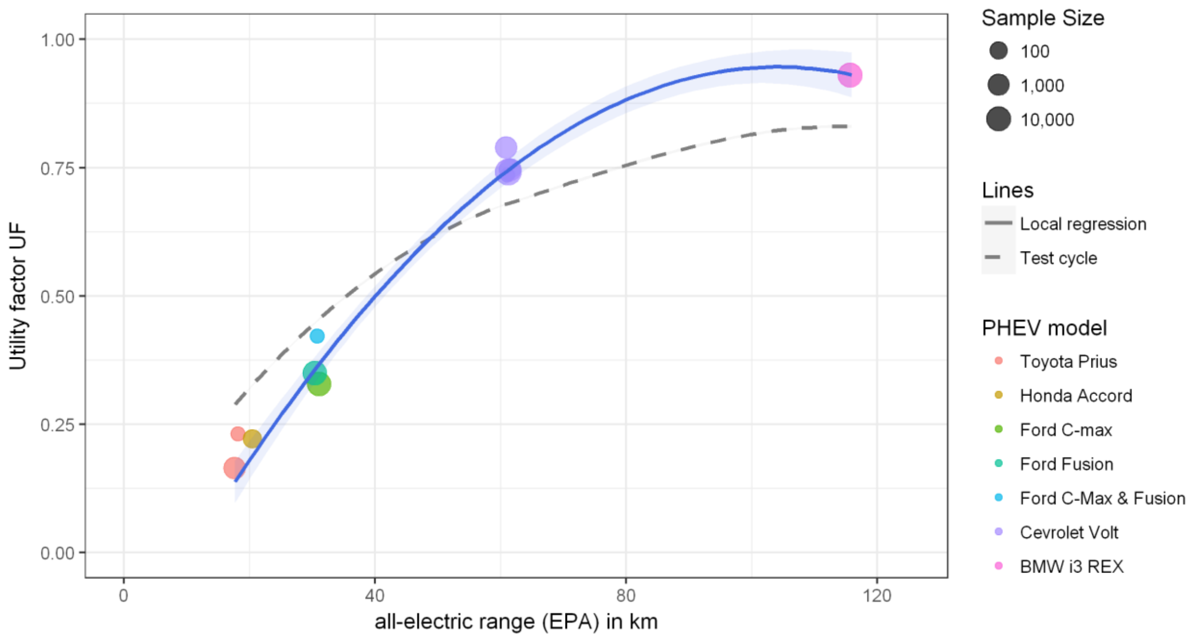

Figure A-1: Utility factors of PHEV in the US with different AER. Shown are mean values per PHEV model with the symbol size indicating the size of the sample as well as a sample size weighted local regression (solid line) and the expectation from EPA tests (dashed line obtained from a regression of the EPA UF values for the PHEV models shown).

German PHEV and BEV data

We based our analysis on German PHEV driving on Spritmonitor.de, a German online web service for car drivers to calculate real-world kilometre cost including all operating cost. This database contains information for different vehicle types, including PHEV (see also Table A-4). Among other information, registered car drivers report their fuel demand in litres and the corresponding cost as well as the vehicle mileage after each refuelling. The resulting average fuel consumption and cost are calculated automatically. Detailed information on distances travelled and the respective fuel consumption for every registered driver are accessible freely on the website. Mock et al.[2] indicate a good representativeness of spritmonitor.de for the German car fleet. For the analysis in the present paper we mainly focus on the PHEV models listed in Table A-5.

In spritmonitor.de average fuel economy for PHEV is reported in two different ways. Most of the users (82.5%) report total fuel economy c_{tot} related to distance driven. Others report average fuel economy in charge sustaining mode c_{cs}. Drivers with more than 30 l per 100 km (below 7.8 MPG) in charge sustaining mode have been removed from the data as outliers. Annual mileage is calculated as extrapolation from the average daily mileage. For drivers that state only their total fuel economy c_{tot} (17.5% of the spritmonitor sample) we use the NEDC value as conservative estimate for their charge sustaining mode fuel economy in order to arrive at an UF. We calculate the user's UF as the difference between unity and the ratio of average c_{tot} and estimated charge sustaining mode fuel economy c_{cs} : $UF = 1 - c_{tot}/c_{cs}$.

Analogously to Figure A-1, the following Figure A-2 summarises the results of the UF of all German PHEV as function of all electric range.

Table A-4: Description of the PHEV database spritmonitor.de.

	**spritmonitor.de**
Available Data	Fuel economy and distance driven between refuelling
Derivable data	Annual mileage, utility factor
PHEV Models and sample size	see Table A-5
Data collection	Fuel quantity and odometer reading after each refuelling reported by driver
Data availability	2007-2017 (PHEV subset)
Fleet structure	Mainly private cars

Table A-5: Summary statistics of the analysed German PHEV models (spritmonitor.de).

PHEV Model	Sample size	NEDC all-electric range in km	UF mean	UF se	UF NEDC
Prius	71	23	35%	2%	48%
C350e	2	31	47%	6%	55%
Porsche Cayenne	2	36	28%	17%	59%
Volvo XC90	4	40	43%	10%	62%
A3 e-tron	27	50	41%	4%	67%
Golf GTE	40	50	40%	3%	67%
V60	19	50	48%	4%	67%
VW Passat	10	50	30%	7%	67%
Outlander	84	52	50%	2%	68%
Ampera	46	83	73%	4%	77%
BMW i3 REX	8	170	83%	6%	87%

Comparative data on German BEV usage was also obtained from spritmonitor.de. Summary statistics are shown in Table A-6.

Table A-6: Summary statistics of the analysed German BEV models (spritmonitor.de).

PHEV Model	Sample size	EPA all-electric range in km	Real world fuel consumption	Yearly VKT
BMW i3	20	130	16.7	18,923
Tesla Model S*	37	335	20.9	34,581
Nissan Leaf	31	135	16.8	16,511
Renault Zoe**	63	158	17.3	16,698
VW eGolf	14	134	16.4	15,582

The distribution of daily VKT has been obtained from the daily VKT of many individual vehicles. Figure A-3 shows the individual distributions of daily VKT for 80 randomly selected vehicles from the PHEV data (all Chevrolet Volt vehicles from the voltstats.net data source) and the overall distribution of full daily driving data set (thick solid line).

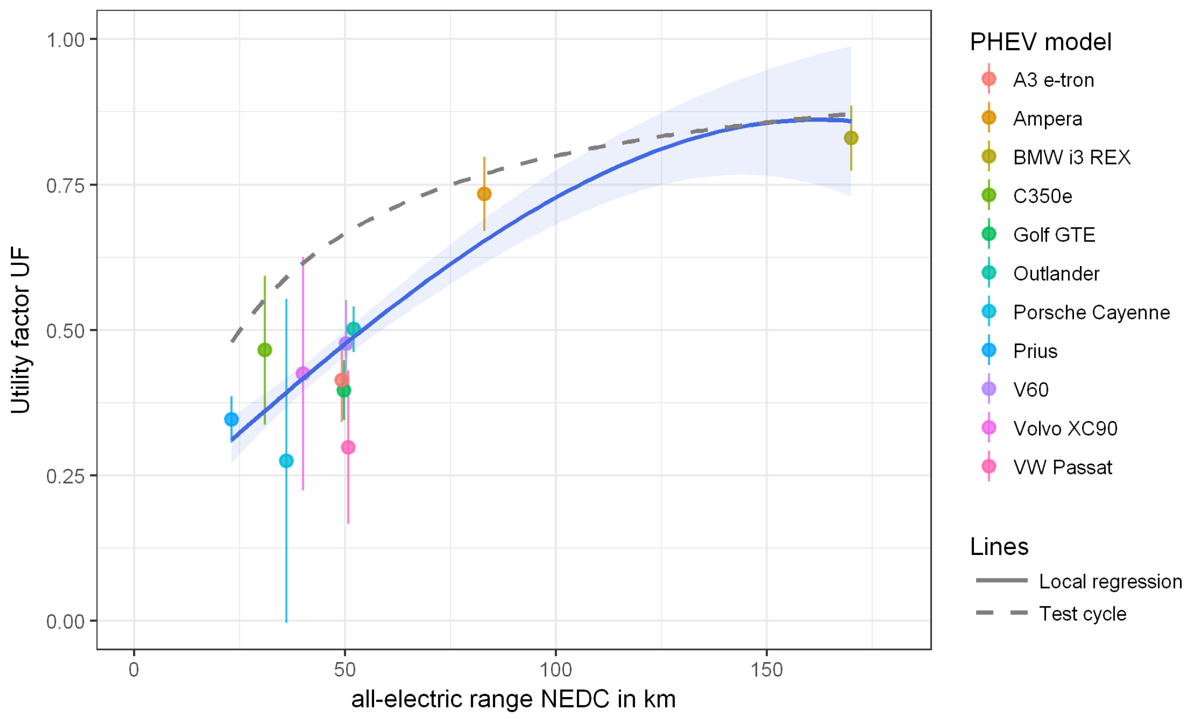

Figure A-2: Utility factors of PHEV in Germany with different AER. Shown are mean values per PHEV model with 2 standard errors as well as local smooth regression (solid line) and the expectation from NEDC test-cycle values given by AER/(AER+25).

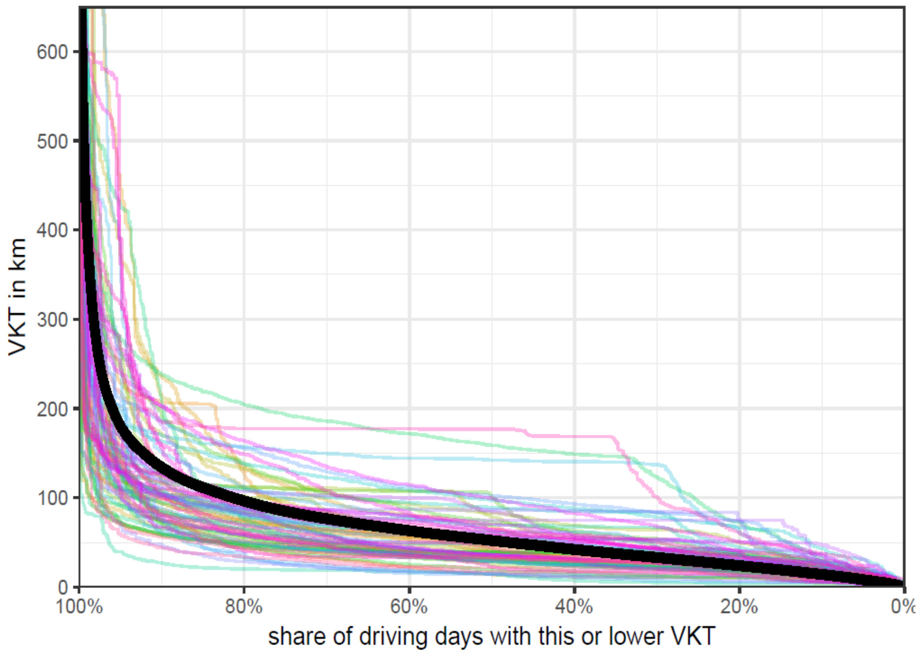

Figure A-3: Distribution of daily VKT for 80 randomly selected vehicles and overall distribution of full daily driving data set (thick solid line).

Methods

All calculations and the local weighted regression analysis has been performed with the R statistical language[3]. More specifically, the local regression lines are locally weighted scatterplot smoothers (LOESS).

Impact on GHG emissions

In the following we focus only on the emission impact when electrifying VKM of passenger cars. We do not consider changes in mobility patterns such as modal shift etc. For estimating the climate impact from electric vehicles two crucial assumption hast to be made. The first is on the emissions of GHG during the production process of the battery and the second is on the caused, indirect GHG emissions during the vehicle usage phase.

The production process of batteries is complex[4] and run through significant improvements during the last years but is still far from being completely matured. This development is already acknowledged from literature and the average emission values are still between 39 – 196 kg CO_{2eq} / kWh[5]. Correspondingly, we assumed for our calculation an additional emission value of 18 kg of CO_{2eq} per km AER. Hence, a kWh of battery capacity causes about 100 kg CO_{2eq} emissions during its production process as stated in the main text. Therefore, the additional emissions for the battery are smaller for PHEV (in average 0.6 t of CO_{2eq}) than for BEV (in average 2.6 t of CO_{2eq}).

However, the internal combustion engine and complex gear box of PHEV is associated with a more complex production process than for a BEV without a gear box. For current passenger cars the share of emissions of CO_{2eq} during the vehicle production phase for the engine and gear box amount to about 20 %[6]. The emissions for the production of an electric motor are still uncertain. We therefore assume the additional emissions for the internal combustion engine of PHEV of about 0.6 t of CO_{2eq} per vehicle. Hence, the CO_{2eq} emissions during the production phase for BEV is reduced by 0.6 t of CO_{2eq} compared to ICEV and PHEV.

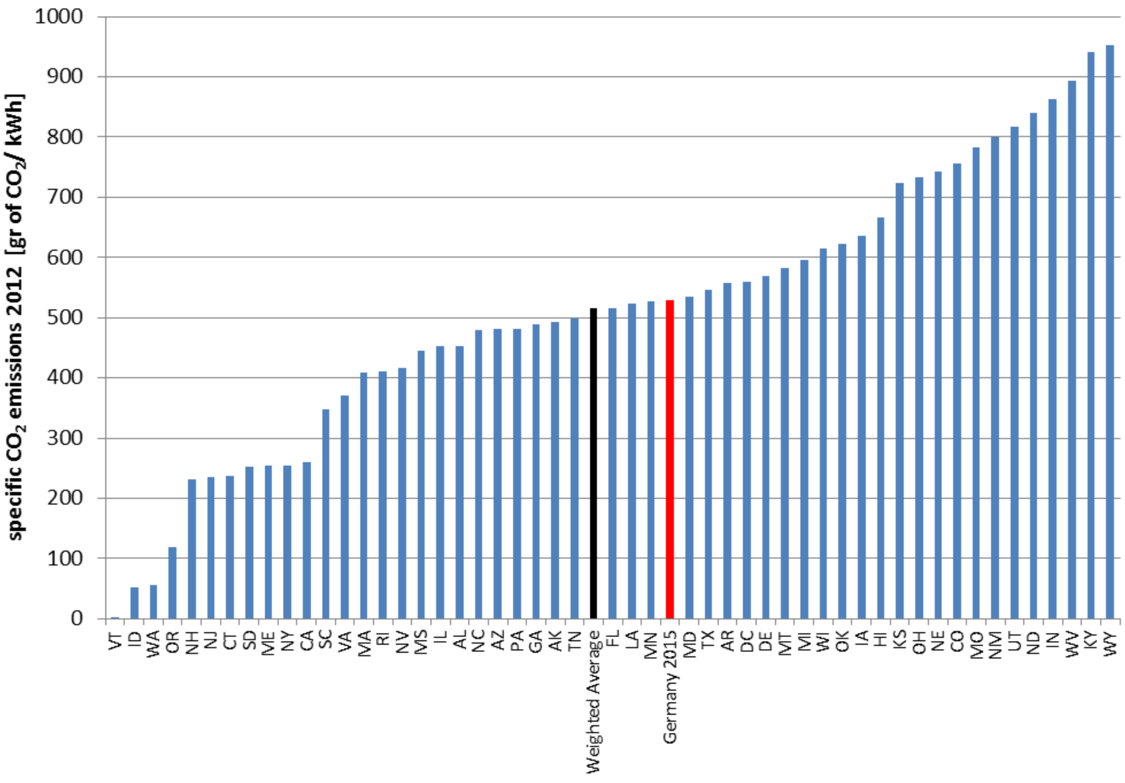

Figure A-4: Specific emissions from electricity generation in US States and Germany for 2012

For the GHG emissions during vehicle usage phase, we choose an average emission based approach[7]. The average GHG emissions per kWh for the German electricity grid were around 0.53 kg of CO_{2eq} per kWh in 2015[8]. In the US, the average values differ strongly between states (cf. Figure A-4). While in Vermont there were only marginal emissions for 2012, the

emissions in Wyoming amount to up to almost 1 kg CO_{2eq} per kWh in the same year[9]. In average, the values show a decreasing trend over time[7,10]. Consequently, we take for our calculations an average emission factor of 0.5 Kg of CO_{2eq} per kWh. This value equals to about 100 g of CO_{2eq} per km when taking an average cross efficiency of 0.20 kWh per km including charging losses. This value is multiplied with the empirical electric VKT from our data sample. For comparing the mitigation of emissions from PHEV and BEV by the battery capacity we multiplied this product with the lifetime of the car and subtracted this value from the estimated emissions which ICEV would have produced (i.e. 120 g CO_{2eq}/km * eVKT * lifetime). The assumption of 120 g CO_{2eq}/km follows those of current registrations that emit 118 g CO_{2eq}/km in official driving cycles[11], which should equal empirical values of around 150 to 165 g CO_{2eq}/km[2] – not including upstream emissions of about 16 g CO_{2eq}/km[12]. Further developments in biofuels might, however, contribute to this optimistic development. The resulting values are given in Figure 4.

For 2030 the advantages for BEV will increase. We therefore sketched an outline of a probable development. We assumed a decrease in GHG emissions for battery production by 50%, an increase of efficiency for BEV to 0.18 kWh/km and to 100 gCO_{2eq}/km for ICEV and an average specific emission value of 290 gCO_{2eq}/kWh[7]. Correspondingly, the BEV would be preferable even from the first electrified AEM in absolute terms. Only in specific terms the advantage of PHEV is still unbeaten, which might be only overcome by a comprehensively established fast charging network, where BEV might increase their eVKM significantly (cf. Figure A-5). We assume that the vehicles usage patterns remain constant as in our dataset.

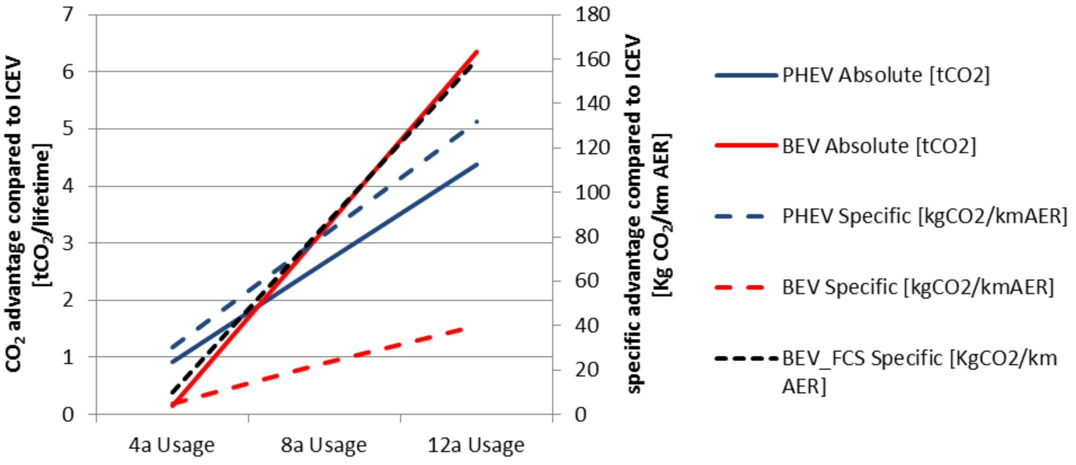

Figure A-5: A scenario for lifecycle advantages of CO_{2eq} emissions from PHEV and BEV compared to conventional vehicles on an absolute scale and relative to battery capacity for 2030.

Table A-7: CO_{2eq} emissions and emission reductions of PHEV and BEV models.

	**AER** [km]	**Electric consumption*** [kWh/100km]	**Additional CO_{2eq} from production** [t of CO_{2eq}]	**CO_{2eq} reduction over lifetime of 8 years** [t of CO_{2eq}]	**CO_{2eq} reduction over lifetime of 8 years per AER** [kg of CO_{2eq}/km_{AER}]
Toyota Prius PHEV	18	15.5	0.32	0.32	17.5
			0.00	0.4	22.5
Ford C-Max Energi	31	23.0	0.56	0.5	15.8
Ford Fusion Energi	31	23.0	0.56	0.6	18.0
Ford C-max/Fusion	31	23.0	0.56	0.7	23.4
Chevrolet Volt (CARB)	61	22.0	1.10	1.25	20.5
			0.00	1.0	16.6
			0.00	1.1	17.9
Honda Accord	21		0.38	0.5	59.4
BMW i3 REX	116	18.0	2.09	0.1	8.7
Nissan Leaf	135	19.0	1.83	0.7	8.1
			1.83	0.8	3.6
Ford Focus Electric	122	20.0	1.60	0.9	0.6
Honda Fit	129		1.72	0.8	5.2
Tesla Model S	335	22.0	5.43	-2.0	2.4
BMW i3	130	17.0	1.74	0.3	6.6

*US-EPA testing, c.f. https://www.fueleconomy.gov/feg/fe_test_schedules.shtml

References Supplement

1. California Air Resources Board, California's Advanced Clean Cars Midterm Review - Summary Report for the Technical Analysis of the Light Duty Vehicle Standards https://www.arb.ca.gov/msprog/acc/mtr/acc_mtr_finalreport_full.pdf (2017).
2. Mock, P., Tietge, U., Franco, V., German, J., Bandivadekar, A., Ligterink, N.E., Lambrecht, U., Kühlwein, J., Riemersma, I., From laboratory to road – a 2014 update of official and "real-world" fuel consumption and CO2 values for passenger cars in Europe, *ICCT White Paper* (2014).
3. R Core Team, R: A language and environment for statistical computing. R Foundation for Statistical Computing, Vienna, Austria. https://www.R-project.org/ (2016).
4. Ellingsen, L. A. W., Majeau-Bettez, G., Singh, B., Srivastava, A. K., Valøen, L. O., & Strømman, A. H., Life cycle assessment of a lithium-ion battery vehicle pack. *Journal of Industrial Ecology*, **18**(1), 113-124 (2014).
5. Kim, H. C., Wallington, T. J., Arsenault, R., Bae, C., Ahn, S., & Lee, J., Cradle-to-Gate Emissions from a Commercial Electric Vehicle Li-Ion Battery: A Comparative Analysis. *Environmental Science & Technology*, **50**(14), 7715-7722 (2016).
6. Daimler, Life cycle. Umwelt-Zertifikat für die E-Klasse http://docplayer.org/14970178-Life-cycle-umwelt-zertifikat-fuer-die-e-klasse.html (2017).
7. Jochem, P., Babrowski, S., & Fichtner, W., Assessing CO 2 emissions of electric vehicles in Germany in 2030. *Transportation Research Part A: Policy and Practice*, **78**, 68-83 (2015).
8. UBA (Umweltbundesamt), Entwicklung der spezifischen Kohlendioxid-Emissionen des deutschen Strommix. Petra Icha, Gunter Kuhs. *CLIMATE CHANGE 26/2016*. https://www.umweltbundesamt.de/sites/default/files/medien/378/publikationen/climate_change_26_2016_entwicklung_der_spezifischen_kohlendioxid-emissionen_des_deutschen_strommix.pdf (2016).
9. EPA, eGRID subregion and GHG emissions finder tool, https://www.epa.gov/sites/production/files/2015-10/power_profiler_zipcode_tool_2012_v6-0_0.xlsx (2017).
10. IEA, World Energy Outlook, https://www.eia.gov/outlooks/ieo/pdf/0484.pdf (2017).
11. EEA, Fuel efficiency improvements of new cars in Europe slowed in 2016: https://www.eea.europa.eu/highlights/fuel-efficiency-improvements-of-new (2017)
12. Moretti, C., Moro, A., Edwards, R., Rocco, M.V., Colombo, E., Analysis of standard and innovative methods for allocating upstream and refinery GHG emissions to oil products, *Applied Energy* **206**, 372-381, doi: 10.1016/j.apenergy.2017.08.183 (2017).
13. Plötz, P., Funke, S.A. and Jochem, P., Empirical fuel consumption and CO2 emissions of plug-in hybrid electric vehicles. *Journal of Industrial Ecology*. doi:10.1111/jiec.12623 (2017).
